# Does the type of surgical attire influence surgical site infection rates in intramedullary nailing for proximal femoral fractures? A retrospective analysis

**DOI:** 10.1007/s00423-025-03889-0

**Published:** 2025-10-31

**Authors:** Maud A. M. Vesseur, Timon van der Burg, Erik R. de Loos, Annette M. Pijnenburg, Wouter L. W. van Hemert, Martijn G. M. Schotanus, Bert Boonen, Raoul van Vugt

**Affiliations:** 1 Faculty of Science and Engineering, Maastricht University, Maastricht, The Netherlands; 2 Department of Orthopedic Surgery, Zuyderland Medical Center, Heerlen, The Netherlands; 3 Department of surgery, division of trauma surgery, Zuyderland Medical Center, Heerlen, The Netherlands; 4 School of Care and Public Health Research Institute, Faculty of Health, Medicine and Life Science, Maastricht University, Maastricht, The Netherlands

**Keywords:** Surgical Site Infections, Superficial Wound Infections, Deep Wound Infections, Surgical Attire, Proximal Femoral Fracture, Sustainability

## Abstract

**Purpose:**

The aim of the study was to examine whether there is an incidence difference on surgical site infections between surgeons using different surgical attire during intramedullary fixation for proximal femoral fractures.

**Methods:**

1,431 patients were included and divided into two groups; surgeons wearing balaclava- or skull caps (490 vs 941). The occurrence of surgical site infection was retrospectively assessed and divided into superficial- and deep wound infections.

**Results:**

The occurrence of superficial wound infections did not differ significantly between the two groups, with three patients in the balaclava and six in the skull cap group (0.6% vs 0.6%, *p* = 1.00). Similarly, there was no significant difference in the occurrence of deep wound infections between the groups, with one case in the balaclava and eight in the skull cap group (0.2% vs 0.9%, *p* = 0.18).

**Conclusion:**

This study found no statistically significant difference in the incidence of surgical site infections (including both superficial and deep wound infections) between balaclava caps and skull caps. These results suggest that the type of surgical attire does not have a significant impact on the occurrence of surgical site infections in intramedullary nailing for proximal femoral fractures. Therefore, factors such as cost, and sustainability should be considered when selecting surgical attire. In this context, the skull cap would be the preferred option.

## Introduction

Hip fractures, a severe consequence of osteoporosis, are becoming increasingly prevalent due to the ageing population. Annually, over 250,000 hip fractures occur in the United States, with projections reaching 4.5 million by 2050 [1]. In Central Europe, falls caused 400,000 hip fractures in 2019 [2]. Prognosis is often poor, with 20–30% of patients deceased within a year after surgery [1, 3]. Surgical site infections (SSI), affecting 2.2% of patients, further increase morbidity, mortality, hospital stay duration, and healthcare costs due to the need for prolonged antibiotic treatment, reoperations, or implant removal [4].

Proximal femoral fractures (PFF) typically require surgical fixation, and the choice of technique depends on various factors including fracture type, patient characteristics, and surgical expertise [5]. Perioperative antibiotic prophylaxis is standard practice to minimize infection risk during surgery, with Cefazolin being a preferred choice in The Netherlands [6]. SSI are of significant concern post-surgery, varying in severity from superficial skin infections to more critical infections affecting deeper tissues and implant [7]. Certain pathogens, like staphylococcus aureus and coagulase-negative staphylococci, are prevalent in implant surgery, necessitating effective preventative measures [8]. SSI risk is multifactorial and extends beyond pharmacologic prevention. The role of the surgical environment, including ventilation systems, sterile techniques, and surgical attire, has gained increasing attention as part of multimodal SSI prevention strategies.

There is a growing focus on preventing SSI, with the debate over different types of surgical attire taking a dominant role. One debated aspect is the choice of surgical head covering, particularly between balaclava caps, which fully cover the head, neck and facial hair, versus skull caps, which leave part of the head and neck exposed, this resulting in varying potential impact on contamination risk [9–13]. Headwear is intended to minimize contamination from hair or skin particles shedding into the sterile field.

Previous studies have compared bouffant caps with skull caps, showing conflicting results and no consistent difference in SSI outcomes [12, 14]. However, direct comparisons between balaclava caps and skull caps are lacking, despite suggestions that balaclava may reduce particle dispersion more effectively, especially among surgeons with long hair or facial hair. Some studies suggest skull caps may offer better material integrity (e.g. lower permeability), but these findings have not been shown to affect clinical endpoints like SSI [12–15]. Current evidence on the impact of cap type is inconclusive, but it remains a source of variation in clinical practice.

Given the absence of definitive evidence and the ongoing debate in surgical guidelines and hospital protocols, comparting the impact of balaclava and skull caps on SSI is of practical and clinical relevance. A clearer understanding may inform future infection control policies. Therefore, this study aimed to evaluate the effectiveness of surgical balaclava caps in preventing SSI, compared to traditional skull caps, in patients with proximal femoral fractures treated with intramedullary nail fixation. It was hypothesized that the use of a balaclava cap would not reduce the incidence of SSI compared to a skull cap.

## Material and methods

### Study design

**Fig. 1:**
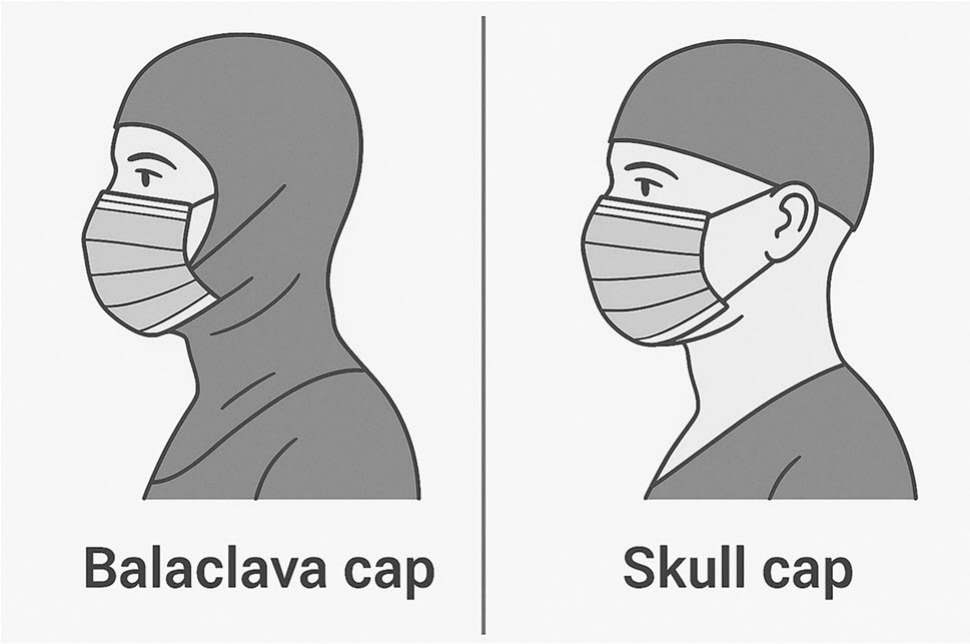
Surgical Attire Description. ^#^ This task/figure was commissioned by M.A.M. Vesseur and completed with the assistance of ChatGPT (OpenAI) on July 27, 2025

This retrospective study compared the incidence of SSI in patients with PFF who underwent intramedullary fixation performed by orthopedic surgeons wearing balaclava cap with those treated by trauma surgeons using skull cap. A schematic illustration comparing balaclava caps and skull caps is presented in Fig. 1. The procedure for intramedullary nail insertion adheres to standardized national guidelines set by the Federation of Medical Specialists in the Netherlands, ensuring uniformity in both groups [16]. Therefore, apart from the surgical cap used, operative procedure and perioperative management were identical between orthopedic and trauma surgeons.

### Outcome measures

This study included patients with PFF classified as AO/OTA 31-A (according to the AO/OTA classification which is a standardized method used to categorize fractures based on their location, type, and severity [17]) indicated for internal fixation who underwent surgery between the 1 st of January 2016 and the 31 st of December 2021 at Zuyderland Medical Center (ZMC), The Netherlands. Exclusion criteria were polytraumatized patients with an Injury Severity Score >15, pathological or periprosthetic fractures, patients who already had an infection (e.g., urinary tract infection, pneumonia) prior to the fracture and patients who already used prophylactic antibiotics at home. Primary outcome was the occurrence of SSI including superficial wound infections (SWI) and deep wound infections (DWI). SSI is defined as an infection that occurs at or near the site of a surgical incision, which is classified into SWI or DWI based on their severity and the layers of tissue involved [18]. Wherein SWI involves the skin and subcutaneous tissue at the incision site and DWI involves also the deeper tissues beneath the skin and subcutaneous layer, such as fascia, muscle or even bone [19]. Secondary outcomes included assessment of costs and generated waste. Data was obtained from ZMC and the manufacturer, Mölnlycke®.

### Data collection

The following variables were retrieved form the patients records: sex, age, fracture type, smoking, alcohol consumption, anticoagulants, time between injury and admission, emergency department date, days between fracture and surgery, surgery duration, responsible department, hospital location, diabetes mellitus, body mass index (BMI), vascular disease, heart/liver/kidney failure, rheumatoid arthritis, chronic glucocorticoids or immunosuppression, dementia or cognitive impairments, invasive devices (e.g., catheters, ventilators), American Society of Anesthesiologist (ASA) score, prophylactic and pre-operative antibiotics, implant type, nail length, open/closed reposition, first operator, post-operative antibiotics, blood transfusion, SWI, DWI, revision surgery, re-hospitalization within 30 days, hospitalization length, time to mobilization, other relevant info (e.g., complications), and discharge disposition.

### Statistical analysis

Variables that could potentially affect SSI are sex, BMI, ASA score, diabetes mellitus, alcohol consumption, fracture type, dislocation or subluxation, incision cleanliness grade, mechanism of injury, chronic heart disease, allergies, and the use of antibiotic prophylaxis [20, 21]. These variables were accounted for in the statistical analysis. A non-inferiority power calculation determined that 1,514 patients (757 per group) were required. This calculation assumed an SSI incidence of 2.2% in PFF, with a control group success rate of 97.8% based on literature. Preserving 2.5% of the control effect, the non-inferiority margin was set at 2.445%. Given no expected difference in SSI outcomes between balaclava cap and skull cap, the experimental group’s success rate was assumed to match the control. To strengthen the study, the dataset was increased to 1,718 patients. Given its retrospective nature, ethical standards remain unaffected by this adjustment. Chi-square tests and Independent T-tests were used to compare categorical and continuous data respectively. Descriptive data included mean and standard deviation (SD) for continuous variables, and number and frequencies for categorical variables to describe patient baseline characteristics, surgery types, and clinical outcomes. *P*-value of <0.05 was considered as statistically significant. Statistical analysis was performed using IBM SPSS Statistics (Version 29, IBM Corp., Armonk, NY, USA) to ensure accurate data processing and analysis [22].

### Ethical approval

The protocol of this retrospective study received full approval from the Medical Ethics Assessment Committee. The file number of the application is METCZ20200127. To ensure quality and transparency this report was written in compliance with the Strengthening in Reporting of Observational Studies in Epidemiology (STROBE) guidelines for cohort studies [23].

## Results

**Fig. 2:**
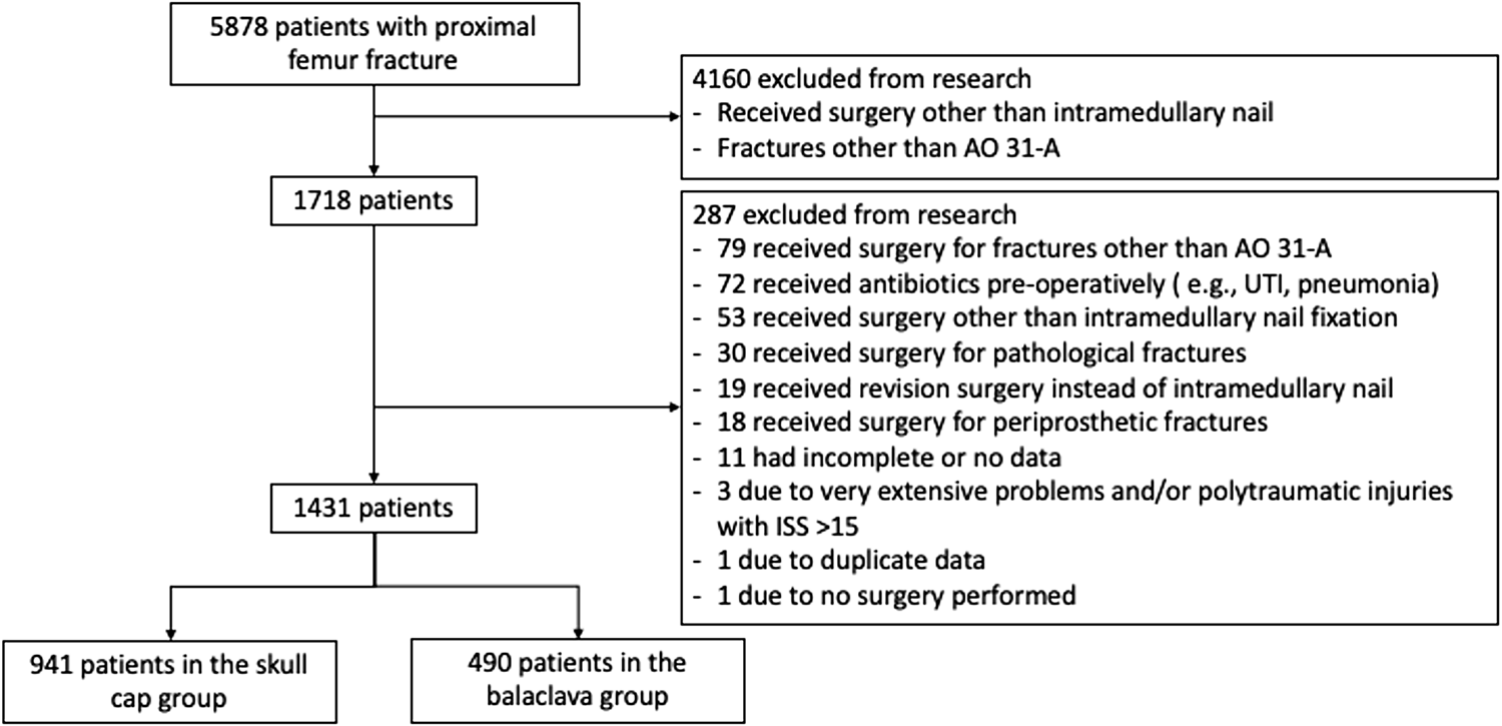
Flowchart

A total of 5,878 patients with PFF were identified. Of these, 4,160 patients were excluded prior to analysis as they did not undergo intramedullary nailing. As a result, a total of 1,718 patients were analyzed of which 1,431 met the inclusion criteria. Reasons for dropout are described in Fig. 2. Demographic data and comorbidities were compiled in Table 1 for a comprehensive assessment of SSI occurrence, considering both SWI and DWI.

**Table 1:** Demographics of study population

Demographics (*n*=1431)		Skull Cap (*n*=941)	Balaclava Cap (*n*=490)	p-value
Age y (mean (SD))		81.22 (10.90)	80.42 (11.37)	0.199
ASA-score (%)	1	74 (7.9)	28 (5.7)	0.397
	2	400 (42.5)	224 (45.7)	
	3	426 (45.3)	216 (44.1)	
	4	41 (4.4)	22 (4.5)	
Current smoker (%)		67 (63.8^1^)	38 (66.7^2^)	0.716
Diabetes mellitus (%)		179 (19.0)	74 (15.1)	0.065
Peripheral vascular disease (%)		77 (8.2)	36 (7.3)	0.578
Chronic therapy with glucocorticoids (%)		50 (5.3)	26 (5.3)	0.995

^*1*^*N* = 105, this is a smaller group since this variable is not well dmented

*N*^*2*^*N* = 57, this is a smaller group since this variable is not well documented

The study identified 18 documented SSI, comprising nine SWI and nine DWI (Table 2), translating into an overall infection rate of 1.26% (95% confidence interval [CI], 0.96%−1.55%). Within the balaclava cap group (*n*=490), four SSI were observed, including three SWI and one DWI, resulting in an overall infection rate of 0.82% (95% CI, 0.02%−1.61%). In the skull cap group (*n*=941), 14 SSI were recorded, with six SWI and eight DWI, producing an overall infection rate of 1.49% (95% CI, 0.71%−2.26%). There was no statistically significant difference between the balaclava cap group and skull cap group for combined wound infections (Pearson Chi-Square, *p*=0.279) (Table 2).

**Table 2:** Total SSI between the Balaclava Cap and Skull Cap

SSI (*n*=1431)		Surgical site infections		Total
		No	Yes	
Skull Cap vs Balaclava Cap	Skull Cap	927	14	941
	Balaclava Cap	486	4	490
Total		1413	18	1431

For SWI, a total of nine infections were documented (Table 3). In the balaclava cap group, three SWI were reported (*n*=490), resulting in an infection rate of 0.61% (95% CI, −0.08%−1.30%). In the skull cap group, six SWI occurred (*n*=941), yielding an infection rate of 0.64% (95% CI, 0.13%−1.15%). No statistically significant difference was observed between the two groups concerning SWI (Fisher’s Exact Test, *p*=1.000) (Table 3). Regarding DWI, a total of nine infections were recorded (Table 4). In the balaclava cap group, one DWI was documented (*n*=490), resulting in an infection rate of 0.20% (95% CI, −0.20%−0.60%). In the skull cap group, eight DWI were observed (*n*=941), with an infection rate of 0.85% (95% CI, 0.26%−1.44%). No statistically significant difference was found between the balaclava cap group and skull cap group in terms of DWI (Fisher’s Exact Test, *p*=0.178) (Table 4). Other variables, such as overweight (BMI >25 kg/m^2^) and peripheral arterial disease, which were compared between the balaclava cap group and skull cap group, were not found to be statistically significant.

**Table 3:** SWI between the Balaclava Cap and SC

SWI (*n*=1431)		Superficial wound infections		Total
		No	Yes	
Skull Cap vs Balaclava Cap	Skull Cap	935	6	941
	Balaclava Cap	487	3	490
Total		1422	9	1431

**Table 4:** DWI between the Balaclava Cap and Skull Cap

DWI (*n*=1431)		Deep wound infections		Total
		No	Yes	
Skull Cap vs Balaclava Cap	Skull Cap	933	8	941
	Balaclava Cap	489	1	490
Total		1422	9	1431

A cost analysis to assess the financial implications of using different types of head caps during surgeries was performed. The cost difference between balaclava cap and skull cap was €0.32 per cap in preference of skull cap. Considering that each surgery was conducted with a new surgical cap. Approximately four caps were used every single operation. With 490 patients operated on by the orthopedics department, this could have potentially resulted in a total cost reduction of €627.20 within a six-year period.

Product datasheets from Mölnlycke® were utilized to evaluate waste generated by different head cap types. balaclava cap weighed 12 grams each, while skull cap weighed 3.6 grams each, representing a 70% reduction in waste for skull cap. Considering 490 patients in the orthopedics department, the study suggested a potential waste reduction of at least 16,464 grams within a six-year period, attributed solely to the use of skull cap [24].

## Discussion

In the medical field, sustainability has gained significant interest, urging a re-evaluation of one time use materials. This shift is especially crucial in surgical settings, where even minor differences in used material can significantly impact the carbon footprint, given the high frequency of procedures. Globally, the healthcare sector contributes to 4–6% of net carbon emissions, with the operating theatre being the major energy and waste consumer. It is imperative to explore strategies to reduces waste production without compromising patient care quality. Therefore, comparing balaclava cap to skull cap is of interest, as balaclava cap produces more waste than skull cap. This considering the escalating healthcare costs driven by various factors like healthcare service prices, healthcare intensity, population growth, and ageing demographics [25–27].

This study aimed to evaluate the effectiveness of surgical balaclava caps in preventing SSI, compared to traditional skull caps, in patients with proximal femoral fractures treated with intramedullary nail fixation. The hypothesis proposed non-inferiority between the two surgical attire types. The primary outcome analysis—SSI incidence—supported the hypothesis, showing no statistically significant difference between balaclava cap and skull cap. Given that both are for disposable use, this raises concerns about environmental and financial impacts.

While there is no previous research directly comparing balaclava cap and skull cap, studies have compared bouffant caps and skull cap. For instance, the study of Shallwani et al. concerning 20,621 patients (spinal- and craniotomy/craniectomy procedures) and the study of Kothari et al. involving 1,543 patients (colon-, intestinal-, hernia-, biliary- and foregut procedures) had already indicated that there was no statistically significant difference in SSI rates between bouffant caps and skull cap [12, 14]. Moreover, the study by Markel et al. revealed that bouffant caps exhibited greater permeability, higher penetration, and larger microbial shedding compared to skull cap. These findings suggested that skull cap may offer better protection against surgical area contamination. But the absence of any significant difference in SSI development between bouffant caps and skull cap contradicts the theoretical advantages of skull cap [12–15].

The statistically significant difference in surgical duration between the orthopedic and trauma surgery departments did not translate into a reduced risk of SSI. While trauma surgeons performed procedures more quickly—likely due to the higher caseload in their department (941 procedures compared to 490 in orthopedics)—this did not correspond with a lower infection rate. Instead, a trend was observed toward an increased risk of DWI in the skull cap group. This finding contrasts with existing literature, which generally associates prolonged operative time with higher infection risk. For instance, Cheng et al. conducted a systematic review of 81 prospective and retrospective studies and reported that each additional 15 minutes of surgery increased the risk of SSI by 13%. In the study by Cheng et al., most studies combined different types of SSI into one overall rate. It is therefore not possible to determine whether this study had the same distribution of different types of SSI [28].

The statistically significant association between time to post-surgery mobilization and SWI raises complex causal questions. Delayed mobilization may result from SSI and its symptoms, or conversely, it may contribute to SSI development. Additionally, patient factors such as impaired immunity or delirium, which could lead to wound interference, might play a role. The clinical significance of this study’s findings remains uncertain, highlighting the need for further research to clarify the direction of this relationship [29].

The cost savings of approximately €627.20, while modest relative to overall healthcare expenditure, highlight opportunities for reassessing expenses. This study serves as a model for other departments, centers, or manufacturers to evaluate material use. Extending beyond the local situation, such savings could apply to other surgical departments across the Netherlands, contributing to significant cost reductions in a system facing rising expenses [25, 26].

In addition, this study roughly estimated potential waste reduction associated with different surgical cap materials, recognizing that manufacturing contributes more waste than the caps themselves. Using data from ZMC and Mölnlycke®, a rough estimation of waste generation was made. Given rising healthcare costs, a preliminary cost analysis was also conducted, acknowledging additional expenses linked to various head cap materials [24–27]. A reduction of 16,464 grams of waste annually, though modest relative to the healthcare sector’s total waste, represents a meaningful step forward. This impact could grow substantially with broader participation across centers and departments. In addition to waste reduction, the choice between disposable surgical skull caps and bouffant caps has implications for environmental impact, particularly concerning carbon footprint. While specific data comparing the carbon footprints of these two disposable headwear types are limited, it is recognized that the healthcare industry significantly contributes to global carbon emissions, with single-use plastics being a notable factor. The production and disposal of disposable medical products, including surgical headwear, add to this environmental burden. Therefore, selecting headwear that reduces material usage, such as lighter-weight skull caps, may contribute to a marginal decrease in overall carbon emissions. However, comprehensive life cycle assessments are necessary to quantify the exact environmental benefits of choosing skull caps over bouffant caps. This study encourages further exploration of waste reduction opportunities, highlighting the need for more comprehensive data and research in this area.

The results of this study emphasize the need for a deeper understanding of the impact of surgical attire. A meta-analysis, by synthesizing data from multiple studies, would provide greater statistical power and generalizability. This approach allows for standardized comparisons of surgical attire types, such as balaclava cap and skull cap, and could yield more robust insights into their influence on SSI rates, guiding evidence-based practice [30].

The study’s retrospective design offered both strengths and limitations. Strengths included its replicability, confounder analysis, and practical applicability. The study’s clear design, defined variables, and transparent inclusion and exclusion criteria enhance its reproducibility. By examining numerous confounders related to SSI development, the analysis was comprehensive and provided actionable insights, paving the way for follow-up studies.

However, limitations inherent to the retrospective study design were evident. Missing data reduced the sample size and statistical power. For example, smoking status was missing in approximately 80% of cases, which limits our ability to adjust for this known risk factor for SSIs. Non-randomly distributed missing data further risked bias. Additionally, reliance on existing documentation without direct oversight posed risks of recall bias, incomplete records, and limitations in controlling confounders due to the absence of randomization and blinding [31–33]. We were unable to account for individual surgeon characteristics such as hair length and presence of facial hair. These may influence both cap choice and contamination risk, and their absence represents a potential source of unmeasured confounding. Altough we performed a power analysis, the final sample size fell slightly short of the required number. Additionally, the unequal group sizes may have affected the power to detect differences between groups and introduced imbalance in covariates. Without further investigation, these results cannot be readily extrapolated to implant cases, highlighting another important limitation.

## Conclusion

This study found no statistically significant difference in the incidence of SSI, including both SWI and DWI, between the use of balaclava caps and skull caps in patients undergoing intramedullary nailing for proximal femoral fractures. These findings suggest that, within the limitations of a retrospective design, the choice of surgical headwear may not have a major impact on SSI rates. Practical considerations such as cost-effectiveness, comfort, and sustainability may therefore guide local policy, though further high-quality prospective studies or meta-analyses are needed to validate these results before firm recommendations can be made.

## Data Availability

No datasets were generated or analysed during the current study.

## Appendix

**Table 5:** Group Heterogeneity Comparison

Variables	Skull Cap group	Balaclava Cap group	P-value (statistical test)	Significant for causing SSI
Heart failure	36.2%	28.6%	.004 (Pearson Chi-Square)	Not Significant (*p*>.05)
Renal failure	16.2%	9.6%	<.001 (Pearson Chi-Square)	Not Significant (*p*>.05)
Length of surgery	39.8 minutes	44.7 minutes	<.001 (Independent T-Test)	Significant with SWI (*p*=.001), not with DWI (*p*>.05)
Length Until Post-Surgery Mobilization	2.38 days	2.14 days	<.001 (Independent T-Test)	Significant with SWI (*p*<.001), not with DWI (*p*>.05)
